# Genomic Correlations Between the Gaits of Young Horses Measured by Accelerometry and Functional Longevity in Jumping Competition

**DOI:** 10.3389/fgene.2021.619947

**Published:** 2021-01-29

**Authors:** Manon Dugué, Bernard Dumont Saint Priest, Harmony Crichan, Sophie Danvy, Anne Ricard

**Affiliations:** ^1^Université Paris-Saclay, INRAE, AgroParisTech, GABI, Jouy-en-Josas, France; ^2^Pôle Développement Innovation Recherche, IFCE, Gouffern en Auge, France

**Keywords:** genomic, longevity, jumping horses, heritability, genetic correlations

## Abstract

Functional longevity is essential for the well-being of horses and the satisfaction of riders. Conventional selection using longevity breeding values calculated from competition results is not efficient because it takes too long to obtain reliable information. Therefore, the objective was to identify early criteria for selection. We assessed two types of early criteria: gait traits of young horses and QTLs. Thus, our aim was to estimate the genetic correlation between gait traits and longevity and to perform a genome-wide association study (GWAS) for longevity. Measurements of gaits by accelerometry were recorded on 1,477 show jumping horses that were 4 to 5 years old. Gait analysis provided 9 principal components describing trot, canter, and walk. Longevity estimated breeding values (EBVs) for stallions were calculated using a survival analysis of more than 900,000 years of performances by 179,448 show jumping horses born from 1981 onwards. Longevity was measured as the number of years spent in competition. Model included region and month of birth, age at first competition, year, and performance level. Longevity EBVs were deregressed to obtain weighted pseudo-performances for 1,968 stallions. Genomic data were available for 3,658 jumping horses. Seventy-eight percent of the horses measured for gaits and twenty-five percent of those measured for longevity were genotyped. A GWAS of longevity revealed no significant QTLs. Genetic parameters between each of the 9 principal components of the gait variables and longevity were evaluated with a bi-trait animal linear mixed model using single-step GBLUP analysis with the relationship matrix constructed from genomic data and genealogy (24,448 ancestors over four generations). The heritability of the gait traits varied from 0.11 to 0.44. The third principal component for trot (high lateral activity) and the first principal component for canter (high dorsoventral activity and low stride frequency) were moderately genetically correlated with higher longevity: r_g_ = 0.38 (0.15) and 0.28 (0.13), respectively. Our study revealed that functional longevity is a polygenic trait with no major genes. We found new correlations between longevity and gait traits. Before using gait characteristics in a selection plan, these correlations need to be understood better at the biomechanical level.

## Introduction

Jumping is one of the most popular equestrian sports in Europe and consequently the main breeding objective of riding horse breeds (Koenen et al., 2004). The welfare and health of the horses are major concerns for riders, and adding durability and longevity in selection plans is now commonplace (Hartig et al., 2013a,b). Longevity is mostly understood as time spent in competition and measured as the number of years in competition (Braam et al., 2011; Jonsson et al., 2014a; Posta et al., 2014; Seiero et al., 2016) or the number of competition tests (Sole et al., 2017). Only Wallin et al. (2000) considered actual lifespan. The main issue in riding horse breeding is the efficiency of breeding values for longevity. Reliable estimates are obtained late in the life of a sire. Many breeders, therefore, use indirect criteria to predict longevity. Traditional evaluations performed during the testing of young horses are the most common traits used after calculation of genetic or phenotypic correlations with the lifetime in sport competition. The main causes of culling are related to locomotion pathologies (Leblond et al., 2000, 2001; Wallin et al., 2000; Stock and Distl, 2005; Sloet van Oldruitenborgh-Oosterbaan et al., 2010; König von Borstel and Bernhard, 2013; Visser et al., 2014). Therefore, analysis of the gaits of young horses may be a relevant precocious indirect selection criterion for longevity. Recent advances in wearable technologies readily allow locomotor performances to be objectively measured in the field using kinematic or accelerometric analysis (see Egan et al., 2019 for a review). Another way to identify early criteria for the selection of horses with a better longevity is through the use of genomic information because it can be available at birth. This is an option when there are QTLs. Moreover, the availability of detailed genomic data can improve the efficiency of genetic analysis, with a more accurate relationship matrix between relatives.

The aim of this study was to use a description of gaits based on accelerometric data and competition results to estimate the genetic correlation between functional longevity and gait characteristics. Genomic data were added to improve the reliability of the results and to search for the presence of QTL for longevity using a genome-wide association study (GWAS).

## Materials and Methods

### Gaits Data

A group of 1,477 show jumping horses aged 4 (42%) to 5 years (58%) were measured during 27 official young horse competitions in France, during the Spring of 2015 and 2016. This group of horses was composed of 50% females, 35% geldings, and 15% males. A large majority of them (83%) were Selle Français. The rest were foreign sport horses (10%), Anglo-Arabs (3%), or crossed saddle breeds (4%). The gaits were measured using an Equimetrix® accelerometer (Barrey et al., 1994) after the competition, while the horses were being ridden.

Recordings were taken by an accelerometer held in place by a leather pocket over the caudal part of the sternum on the girth. The riders were asked to perform a quick gait test with walk, trot, and canter in an arena that was 60 meters across in order to have a sufficiently long duration of measurement. A 10-s sample of the gait recording was extracted for analysis. This sample corresponded approximately to the time spent in the diagonal of the arena and it was selected manually by visualization of the dorsoventral acceleration using the software provided by Equimetrix®. Four observers selected the samples. Vertical, longitudinal, and lateral accelerations were recorded for five gaits: walk, working trot, medium trot, working canter, and medium canter. Eight measurements were taken from the recovered signals for each of the five gaits of the horse: velocity, frequency of strides, regularity of strides, symmetry (except canter), dorsoventral displacement, dorsoventral, longitudinal, and lateral activities. The calculation methods have been described in Barrey et al. (1994), Barrey et al. (1995), and Leleu et al. (2004), and elementary statistics are provided in the Supplementary Material S1.

According to Ricard et al. (2020), each measurement for medium and working gaits was considered as the same repeated genetic trait. Therefore, we retained 3 gait traits: walk, trot, and canter. The measurements were then summarized by the estimated independent random effect of the horse in a mixed model including the fixed effects of gender (3 levels: female, male, gelding), age (4 vs. 5 years old), velocity (except for velocity itself), event (26 levels), and type of gait (working/medium). Finally, principal component analysis was performed gait by gait for these 8 estimated horse effects (7 for canter). For each gait, 3 principal components were retained. This allowed these 38 traits to be synthesized into nine variables (three for walk, three for trot, three for canter).

The height at withers, which is a morphological trait, was provided by the Institut Français du cheval et de l'équitation (IFCE) for 97% of the horses (1,428). This is the parameter that is formally registered, mainly during breeding shows, generally at 3 years of age.

### Longevity in Competition Data

Competition data was provided by the French Equestrian Federation. These data related to horses born from 1981 onwards and that had participated in show jumping competitions between 1985 and 2018. Horses for which a career in show jumping was not the main objective were excluded from the analysis. These were horses participating in eventing or dressage events (for more than a year), horses born abroad or that had started their career with foreign riders, and ponies or draft horses. Ultimately, 179,448 horses remained for the analysis, corresponding to 918,667 cumulative years of performance. The success of a year's performance is characterized by the sum of the points earned. The points depend on the rank and difficulty of the event. All horses that enter an event earn points. Points are allocated on an exponential scale and then log-transformed to achieve a more normal distribution. The raw scale was arbitrary, after standardization the mean was 0, the standard deviation 1, the minimum −4.6, the maximum 4.5, the skewness −0.30, and the kurtosis −0.04.

Among these horses, there were 49% females, 16% males, and 35% geldings. They were born between 1981 and 2014, 25% were born before 1991, 50% were born between 1991 and 2007, and 25% were born after 2007. The Selle Français breed was the most represented, accounting for 77% of the horses, followed by 7% Anglo-Arabs, 4% French Trotters, 3% Thoroughbreds, 1% foreign sport horses, 4% crossed breed horses, and 1% horses with unknown origins.

Longevity was measured based on the number of years in competition. This equated to the sporting lifetime. It was calculated as the difference between the last year and the first year of competition. Thus, it could include the years without any competition entries. The lifetimes of horses still in competition in 2018 were considered to be censored. Twenty-two percent of the data was censored (39,646 horses).

### Genomic Data

Some of the horses with gait data were genotyped, as were other jumping horses and stallions derived from previous studies (Ricard et al., 2013; Chassier et al., 2018). A total of 3,658 jumping horses were genotyped with three different SNP density chips: the Illumina Equine SNP50 BeadChip (54 k) that includes 54,602 SNPs, the Illumina Equine SNP70 BeadChip (65 k) that includes 65,157 SNPs, and the Affymetrix Axiom Equine genotyping array (670 k) that includes 670,806 SNPs. There were 1,157 horses with gaits data that had been genotyped, and 498 horses with longevity data that had been genotyped. Of the 3,658 genotyped horses, 29% were genotyped with the Illumina Equine SNP50 BeadChip, 2% with the Illumina Equine SNP70 BeadChip, and 69% with the Affymetrix Axiom Equine genotyping array. Imputation was performed using Fimpute 3.0 software (Sargolzaei et al., 2014). Equine 3.0 reference sequence (www.ncbi.nlm.nih.gov/assembly/GCF_002863925.1) was used as the reference map when the SNP's position was available or Equine 2.0 (Wade et al., 2009) when it was not available. First, quality control removed SNPs that did not reach the test requirements for quality. The total number of SNPs on the three chips was 675,475. After deleting the SNPs with unknown chromosome locations, 634,148 SNPs remained. We only used autosomal SNP, and as there were 27,731 SNPs on the X chromosome and 2 SNPs on the Y chromosome, 606,415 SNPs remained, which after suppression of a handful of duplicates became 606,343 SNPs. We deleted SNPs with a MAF < 5% (196,864), followed by the Hardy-Weinberg disequilibrium test with P < 10^−6^ (25,596), a call rate < 90% (6,614), and then for inconsistency between the MAF of the different chips with P<10^−5^ (1,582). Ultimately, 375,687 SNPs remained: 9.9% were on the Illumina Equine SNP50 chip, 11.9% on the Equine SNP70 BeadChip, and 99.5% on the Affymetrix Axiom Equine chip. Imputation was performed on all of these SNPs, adding the information of pedigree data for over four generations (18,682 horses). Details of the imputation process were described by Chassier et al., 2018, who used the same data, supplemented here with 1,241 additional horses genotyped with the Affymetrix Axiom Equine genotyping array.

### Pedigree Data

The pedigree data was provided by the Institut Français du cheval et de l'équitation, on behalf of the breeding organizations.

The study of longevity involved sire and maternal grandsire relationships. The 179,448 horses with measured longevities were derived from 8,991 sires and 9,477 maternal grandsires. The ancestors of these sires represented 16,564 stallions included in the survival analysis to construct the relationship matrix. After the longevity analysis, the sires used to perform join analysis with the accelerometric data were restricted to those with sufficient accuracy. Hence, only 1,968 stallions were retained at the end of the longevity analysis. The ancestors of these horses, those of the 1,477 horses with gaits, and those of the 3,658 genotyped horses were reassembled over four generations, and they constituted a group of 24,446 horses. The horses with gait performances were derived from 486 sires, which corresponded to an average of three progeny per sire.

### Relationship Between Gait Data, Longevity Data, and Genomic Data

Of the horses with gaits data, 1,157 (78%) were genotyped. Of the 1,968 horses measured for functional longevity, 498 (25%) were genotyped. None had both longevity and gaits data since longevity records were analyzed using a sire model. Of the 1,477 horses with gaits data, 1,035 (70%) had their sire estimated for longevity (224 sires) and 939 (64%) had their sire genotyped (263 sires), namely 782 (53%) with their sire estimated for longevity and genotyped.

### Survival Analysis

The sporting lifetime analysis model was a survival analysis. Choice of the model was based on previous literature (Ricard and Fournet-Hanocq, 1997; Ricard and Blouin, 2011). This survival analysis modeled the hazard function:

$$\lambda \;\left(t,\;z_{i}\right)=\lambda_{0}\;\left(t\right)\;e^{z_{i}^{\prime \;}\;\beta }\Rightarrow \lambda \;\left(t^{j},\;z_{i}\right)=1-\alpha_{j}^{\exp\left(z_{i}^{\prime }\;\beta \right)}$$

with β as the vector of the explanatory variables, *z*_*i*_ as the design vector for horse *i*, $t^{j\;}\in \left[a_{j-1},a_{j}\right]$ as the time of culling or censuring during the year *j*, and:

$$\begin{array}{l} \alpha_{k}=\exp\left(-\int_{a_{k-1}}^{a_{k}}\lambda_{0}\left(x\right)dx\right) \end{array}$$

with λ_0_(*x*) as the baseline hazard function. The survival function was

$$S\left(t^{j},\;z_{i}\right)=\prod_{k=1}^{j-\;1}\;\alpha_{k}^{\exp\left(z_{i}^{\prime }\beta \right)}.$$

The fixed effects were the region of birth (13 levels, administrative regions in France), the month of birth (8 levels, from January to July by month and August to December lumped together), the year of performance (34 levels, from 1985 to 2018), the age at the first competition [6 levels, at 4, 5, 6, 7, 8, and 9 (or more) years old], and the performance level (87 levels). The performance level was determined according to the rank of the year of performance of the horse. The first year, only three levels were used according to the horse's gender (female, male, or gelding). The second year, the performance level was determined by the z-score of the logarithm of the sum of the points obtained the previous year from−2 and lower to +1.0 and higher by steps of 0.5 combined with the three genders (24 levels). The third year, a category was added for horses without points the previous year (always combined with gender, 27 levels). For the fourth year, the range of z-scores was from−2 and lower to +2.0 and higher (33 levels). Random effects were the sire effect and the maternal grandsire effect. The variance-covariance matrix included a relationship matrix built with the 16 564 ancestors. Variance components and effects were estimated by maximizing the joint posterior density using ‘the Survival Kit' software (Meszaros et al., 2013). The heritability was computed using $h^{2}=\frac{4\sigma_{s}^{2}}{\sigma_{s}^{2}+1/p}$, with *p* the proportion of uncensored records and $\sigma_{s}^{2}$ the variance of the sire effect (Yazdi et al., 2002).

### Deregression of Estimated Breeding Values for Longevity

Gait variables follow linear models. Longevity follows a nonlinear survival model. There is currently no software that allows estimation of the genetic correlations between these two types of variables while respecting the model of each one. To analyze them simultaneously, we chose to use the estimated breeding values (EBVs) with the survival model as a measure of longevity. However, these breeding values have been estimated with a variable degree of accuracy. Deregressed EBVs were then the right choice to calculate the pseudo-phenotype for longevity. The pseudo-phenotype y^*^ and weights *w* must follow the simplified linear model

$$\begin{array}{l} {\text{y}}^{*}=\text{u}+\epsilon \end{array}$$

with **u** as the vector of genetic values of the stallion for longevity and **ϵ** as the vector of residuals. The variance-covariances matrices were $V\left(\text{u}\right)=\text{A}\sigma_{u\;}^{\;2}$ and ***V***(**ϵ**) = **D**, with **A** the relationship matrix between sires and **D** a diagonal matrix of the terms $\left(\frac{\sigma_{\epsilon }^{2}}{w_{i}}\right)$ for the i^th^ animal. The BLUP (best linear unbiased prediction) of breeding values according to this model are solutions of

$$\begin{array}{l} \left({\text{D}}^{-1}+\frac{1}{\sigma_{u}^{2}}{\text{A}}^{-1}\right)\left(\hat{\text{u}}\right)=\left({\text{D}}^{-1}{\text{y}}^{*}\right) \end{array}$$

The pseudo-phenotypes were therefore

$$\begin{array}{l} {\text{y}}^{*}=\text{D}\left({\text{D}}^{-1}+\frac{1}{\sigma_{u}^{2}}{\text{A}}^{-1}\right)\left(\hat{\text{u}}\right) \end{array}$$

In this equation, estimates of genetic variance $\left(\sigma_{u}^{2}\right)$ and breeding values (û) were obtained by the survival analysis. The inverse of the relationship matrix can be constructed from genealogies. Matrix **D**, however, was not known. We knew the standard errors of $\hat{\text{u}}$ from the survival analysis, and thus diagonal terms of the inverse of $\left({\text{D}}^{-1}+\frac{1}{\sigma_{u}^{2}}{\text{A}}^{-1}\right)$. Indeed, *V*(û_*i*_ − *u*_*i*_) = *t*_*i*_, by noting *t*_*i*_ as the diagonal term of the inverse for animal *i*, and reliability *R* is defined by $R_{i}=\frac{V\left({û}_{i}\right)}{V\left(u_{i}\right)}=\frac{V\left(u_{i}\right)-V\left({û}_{i}-u_{i}\right)}{V\left(u_{i}\right)}=1-\frac{1}{\sigma_{u}^{2}}t_{i}.$ But it is not easy to link diagonal terms of this inverse to diagonal terms of **D**, i.e., weights *w*_*i*_. On the other hand, it would be easy to connect diagonal terms of the inverse of $\left({\text{D}}^{-1}+\frac{1}{\sigma_{u}^{2}}\text{I}\right)$ to weights *w*_*i*_ because the matrix is then diagonal and readily invertible. The diagonal terms of this inverse correspond to standard errors of $\hat{\text{u}}$ estimated without considering relationships between stallions, but only from information provided by their progeny, grand progeny, etc. with longevity measured in competition. By knowing the standard errors of all EBVs of the stallions, it was possible to deduce those that would be obtained without these relationships thanks to the formula of Harris and Johnson (1998). This formula links the reliabilities (*R*) of EBVs depending on the source of information. We implemented a program (R software) that allowed:

- calculation of *R* of the stallions by deleting information provided by the relationship between them (Ricard et al., 2013), see Supplementary Material S2 for details. Cleaned reliability is $R_{i}^{{}^{\prime }}\;$.
- deduction of weights *w*_*i*_, from $R_{i}^{{}^{\prime }}=1-\frac{1}{\sigma_{u}^{2}\left(\frac{w_{i}}{\sigma_{\epsilon }^{2}}+\;\frac{1}{\sigma_{u}^{2}}\right)}$
- deduction of pseudo-phenotypes y^*^.

In order to retain only consistent pseudo-phenotypes, we calculated the pseudo-phenotypes of the stallions that still had enough reliability left after cleaning. We, therefore, calculated a pseudo-performance for longevity for the stallions with reliability still greater than or equal to 0.40, which corresponded to 1,968 stallions. Selected threshold for reliability (0.40) corresponded to usual mean of reliability qualified as “intermediary” on the publication of breeding values for stallions in France for jumping competition.

### Estimation of Genomic Correlations Between Gaits and Longevity

Genomic correlations were estimated using several bi-trait analyses, each with one of the gait principal components and the longevity pseudo-phenotype.

The nine gait traits, i.e., the principal components of the accelerometric data, followed the similar mixed linear model

$$\begin{array}{l} \text{y}=\text{X}\beta +\text{Z}\text{u}+\text{W}\text{p}+\;\epsilon \end{array}$$

where **y** was the vector of performances (the 9 principal components), **X**, **Z, ** and **W** were design matrices, **β** was the vector of fixed effects, **u** was the vector of random effects of genetic values, and **p** was the vector of random effects of the permanent environment vector (only for trot and canter, for which two recordings were made), and finally, **ϵ** was the vector of residuals. The fixed effects were the competitions during which performances were recorded (26 levels), gender (3 levels, namely female, male, and gelding), and age (2 levels, namely 4 and 5 years). The covariates were velocity and height at withers.

The longevity pseudo-phenotypes were modeled by the simplified linear model

$$\begin{array}{l} {\text{y}}^{*}={\text{u}}^{*}+\;\epsilon^{*} \end{array}$$

where y^*^ was the vector of the pseudo-phenotypes, u^*^ was the vector of the random effects of genetic values, and ϵ^*^ was the vector of residuals.

Variance-covariance matrices between random effects of the models of the two variables were then

$$\begin{array}{l} V\left[\begin{array}{c} \text{u}\\ {\text{u}}^{*} \end{array}\right]=\text{G}⊗\text{H} \end{array}$$

and

$$\begin{array}{l} V\left[\begin{array}{c} \epsilon \\ \epsilon^{*} \end{array}\right]=\left[\begin{array}{cc} \text{R} & 0\\ 0 & {\text{R}}^{*} \end{array}\right] \end{array}$$

with

$$\begin{array}{l} \text{G}=\left[\begin{array}{cc} \sigma_{u}^{2} & \sigma_{uu^{*}}\\ \sigma_{uu^{*}} & \sigma_{u^{*}}^{2} \end{array}\right] \end{array}$$

and

$$\begin{array}{lll} \text{R} & = & \text{I}\sigma_{\epsilon }^{2}\\ {\text{R}}^{*} & = & \text{D}\sigma_{\epsilon^{*}}^{2}\; \end{array}$$

where **D** was defined previously.

The relationship matrix between genetic values **H** was constructed using both genealogy data and genomic data using a single-step GBLUP method (Aguilar et al., 2010; Christensen and Lund, 2010; Fernando et al., 2014; Legarra et al., 2014). Construction and inversion of the **H** was performed using GBLUPF90 (Aguilar et al., 2018). This inverse was then used with ASReml software version 4.1 (Gilmour et al., 2014) to estimate the variance-covariance components by restricted maximum likelihood. ASReml software allows some of these variance components to be fixed. Because genetic and residual variances for longevity were better estimated with the survival analysis, these components were fixed, while the variances for gait traits and covariances between gait traits and longevity were estimated.

### GWAS of Longevity

As a by-product of the single-step approach, we performed a genome-wide association study (GWAS) of longevity with back solutions of SNP effects and *P* values calculated as described in Aguilar et al. (2019). The BLUPF90 family of programs package was used with single-trait analysis of longevity as pseudo-phenotypes with weights. The model was the same as the one above for the bi-trait analysis using all of the genotyped and phenotyped animals for longevity in a single step GBLUP. The H matrix was constructed using preGSf90 and then postGSf90 was used to obtain estimates and *P* values for the SNP effects.

## Results

### Principal Component Analysis of Gaits

Although principal component analysis of gait variables was already reported in Ricard et al. (2020), it is essential for being able to understand the present results. The first three principal components represented 63.9% of the variance for walk, 81.7% for trot, and 88.9% for canter. Table 1 shows the eigenvectors of the three components retained for each gait. Trot and canter had similar variance components: the first one represented the dorsoventral activity negatively associated with stride frequency; the second one for trot and third for canter represented longitudinal activity; the third one for trot and the second one for canter represented lateral activity. For walk, the decomposition was different. The first component represented dorsoventral activity associated with regularity and symmetry but not stride frequency. The second one associated stride frequency with longitudinal activity. The third one represented only symmetry.

**Table 1:** Eigenvectors of the principal component analysis of the standardized variables derived from accelerometric analysis of gaits.

	**Walk**			**Trot**			**Canter**		
**Variable**	**PC1^a^**	**PC2^a^**	**PC3^a^**	**PC1^a^**	**PC2^**1**^**	**PC3^a^**	**PC1^a^**	**PC2^a^**	**PC3^a^**
Velocity	0.00	0.00	0.08	0.05	0.05	0.06	−0.02	0.04	0.09
Stride frequency	0.00	0.61	0.05	−0.45	0.29	0.39	−0.53	0.09	0.55
Regularity	0.45	−0.21	−0.19	0.00	−0.06	−0.06	0.03	−0.05	−0.01
Symmetry	0.29	−0.19	0.93	0.00	−0.02	−0.02			
Dorsoventral displ.^b^	0.60	−0.10	−0.21	0.68	−0.37	−0.05	0.01	−0.01	−0.01
Dorsoventral activity	0.56	0.22	−0.12	0.34	−0.06	0.45	0.78	−0.12	0.06
Longitudinal activity	−0.01	0.56	0.16	0.38	0.83	−0.38	0.31	0.02	0.82
Lateral activity	0.22	0.43	0.05	0.29	0.29	0.70	0.14	0.99	−0.06

a *PC, Principal component*.

b *Dorsoventral displacement*.

### Survival Analysis

**Figure 1:**
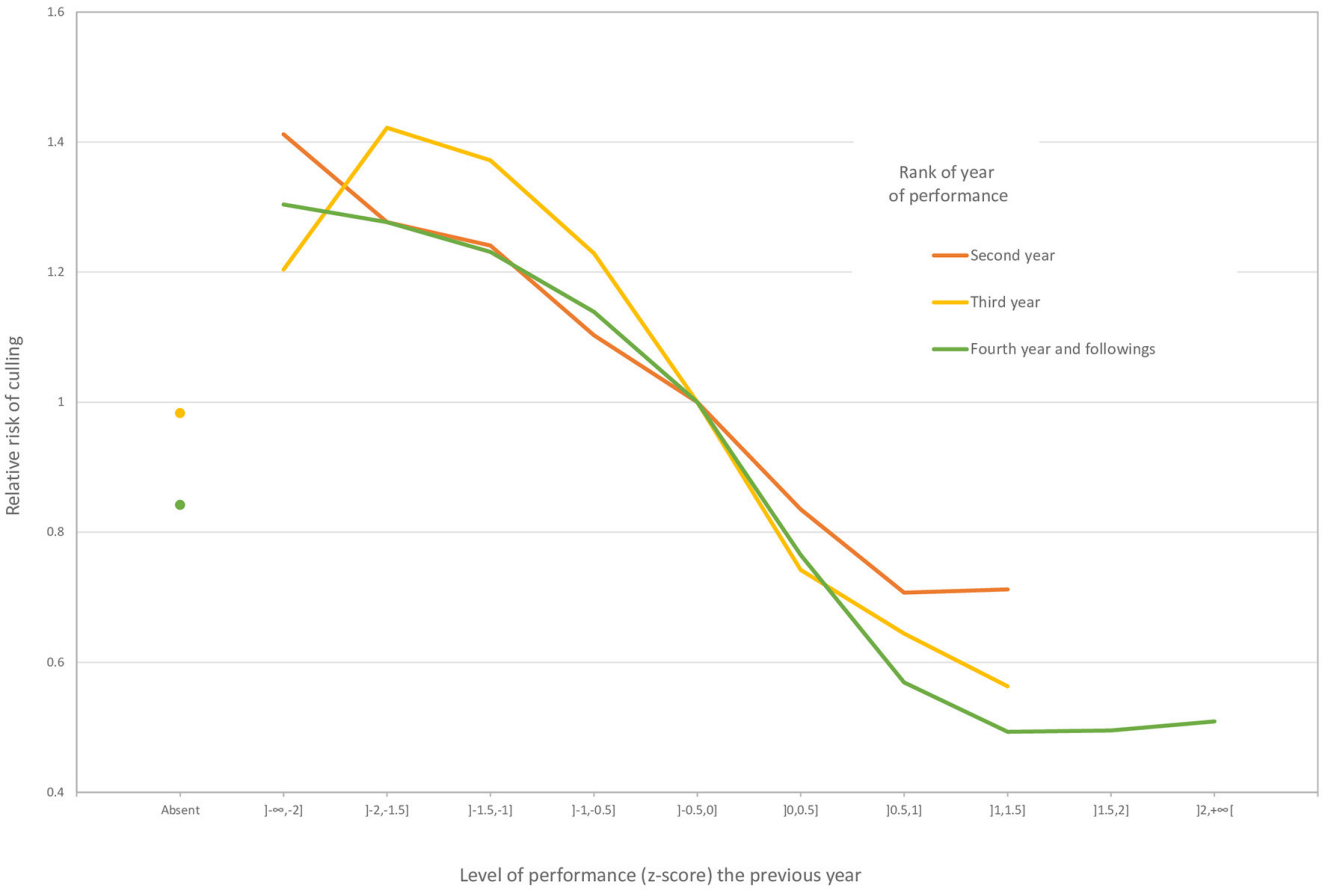
Effect of the level of performance on the risk of culling in jumping competition for geldings.

The sire variance component was estimated by the survival analysis to be 0.03947, which means a heritability equal to 0.12. The main effects concerned the year of performance, the age at the first performance, and the level of performance. The reference for a risk ratio of 1 was a horse born in May in the Normandy region that started competing at 4 years of age in 2006 and that always had average performances (z-score between −0.5 and 0). The risk ratio was higher (> 1.4) for the years between 1985 and 1991 and in 2017. The higher risk ratio for 2017 was a border effect because horses removed temporally from the competition in 2018 did not have the opportunity to come back. The risk of being culled increased with the age at the first performance. Starting competition at the age of four (the reference) had the lowest risk, and this risk was already 1.4 times higher at 6 years of age. The effect of the level of performance is illustrated in Figure 1. Females always had a higher risk of being be culled as males for the same level of performance and geldings at any year of performance. Males mostly followed the pattern of geldings, but the good performers (z-score ≥ 0) had a lower risk of being culled than geldings during the second and third years of performance.

### Pseudo-Phenotypes

The estimates of sire effects for survival analysis and those retained for the calculation of pseudo-phenotypes are summarized in Table 2. After removing information from relatives included in the relationship matrix obtained from the 16,564 ancestors, only 64% of the sires had a reliability higher than 0.01. For the calculation of pseudo-phenotypes for longevity, we chose to keep those with a reliability higher than 0.40, i.e., 1,968 horses (12%). The retained horses amounted to a slightly selected sample, biased for the better sires in terms of longevity (lower risk) but with still a high degree of variability (standard deviation of the relative risk of the sample close to that of the whole population of sires). The corresponding minimum weight was 5 and the maximum was 272 (reliability of 0.97).

**Table 2:** Statistics on the estimated sire effects for functional longevity in jumping competition and for sires retained for the calculation of pseudo-phenotypes.

	***N***	**Mean**	**SE**	**Min**	**Max**
Estimated sire effect	16,564	−0.024	0.133	−0.563	0.617
Estimated relative risk	16,564	0.985	0.131	0.570	1.854
Reliability of sire effects	16,564	0.217	0.217	0.000	0.978
Reliability after pruning relatives	16,564	0.135	0.210	0.000	0.974
**Retained sires**					
Reliability of sire effects	1,968	0.672	0.132	0.421	0.978
Reliability after pruning relatives	1,968	0.625	0.152	0.404	0.974
Estimated relative risk	1,968	0.909	0.138	0.570	1.618
Pseudo–phenotypes	1,968	−0.244	0.470	−1.690	3.204
Weights	1,968	19.795	24.315	5.000	271.827

### Genomic Correlation Between Gaits and Longevity

The results of the estimates of genetic correlations (r_g_) between longevity and each of the gait traits are provided in Table 3, as are the estimated heritabilities for each of the gait traits. For the third principal component of walk, no results could be found because there was no convergence due to the low heritability of this trait (0.04). The heritability of the gait traits varied from 0.11 for canter PC2 related to lateral activity to 0.44 for trot PC1 related to stride frequency against dorsoventral displacement. The repeatability (ratio of the sum of the permanent environment effect variance and genetic variance on phenotypic variance) for trot and canter was very high for all PCs between the two measurements taken at medium and working gaits: from 0.45 to 0.74. A negative correlation between longevity and another trait means that this trait has a positive impact on longevity, as the genetic values of longevity correspond to a risk ratio. The higher the EBV for longevity, the worse the longevity. Two genetic correlations were significantly different from zero. The third principal component of trot, linked to lateral activity, was moderately correlated with longevity: r_g_ = −0.38 (SE 0.15, *P* value = 0.01). The first principal component of canter was moderately correlated with longevity: r_g_ = −0.28 (SE 0.13, *P* value = 0.03). This principal component is linked to dorsoventral activity against stride frequency.

**Table 3:** Heritability, repeatability of gait traits and height, and genomic correlation with functional longevity.

	**Gait traits**		**Genomic correlation**
	**Heritability**	**Repeatability**	
Walk PC1	0.21*** (0.06)		0.24 (0.16)
Walk PC2	0.11*** (0.04)		0.23 (0.20)
Trot PC1	0.44*** (0.06)	0.73*** (0.01)	−0.12 (0.11)
Trot PC2	0.26*** (0.05)	0.63*** (0.02)	−0.11 (0.13)
Trot PC3	0.17*** (0.04)	0.45*** (0.02)	−0.38** (0.15)
Canter PC1	0.28*** (0.05)	0.55*** (0.02)	−0.28* (0.13)
Canter PC2	0.11*** (0.04)	0.46*** (0.02)	0.00 (0.19)
Canter PC3	0.16*** (0.05)	0.45*** (0.02)	−0.02 (0.16)
Height at withers	0.38*** (0.06)		0.08 (0.13)

SE in parenthesis, *** represent P < 0.001, ** represent P < 0.01, * represent P < 0.05.

### Genomic Analysis of Longevity

**Figure 2:**
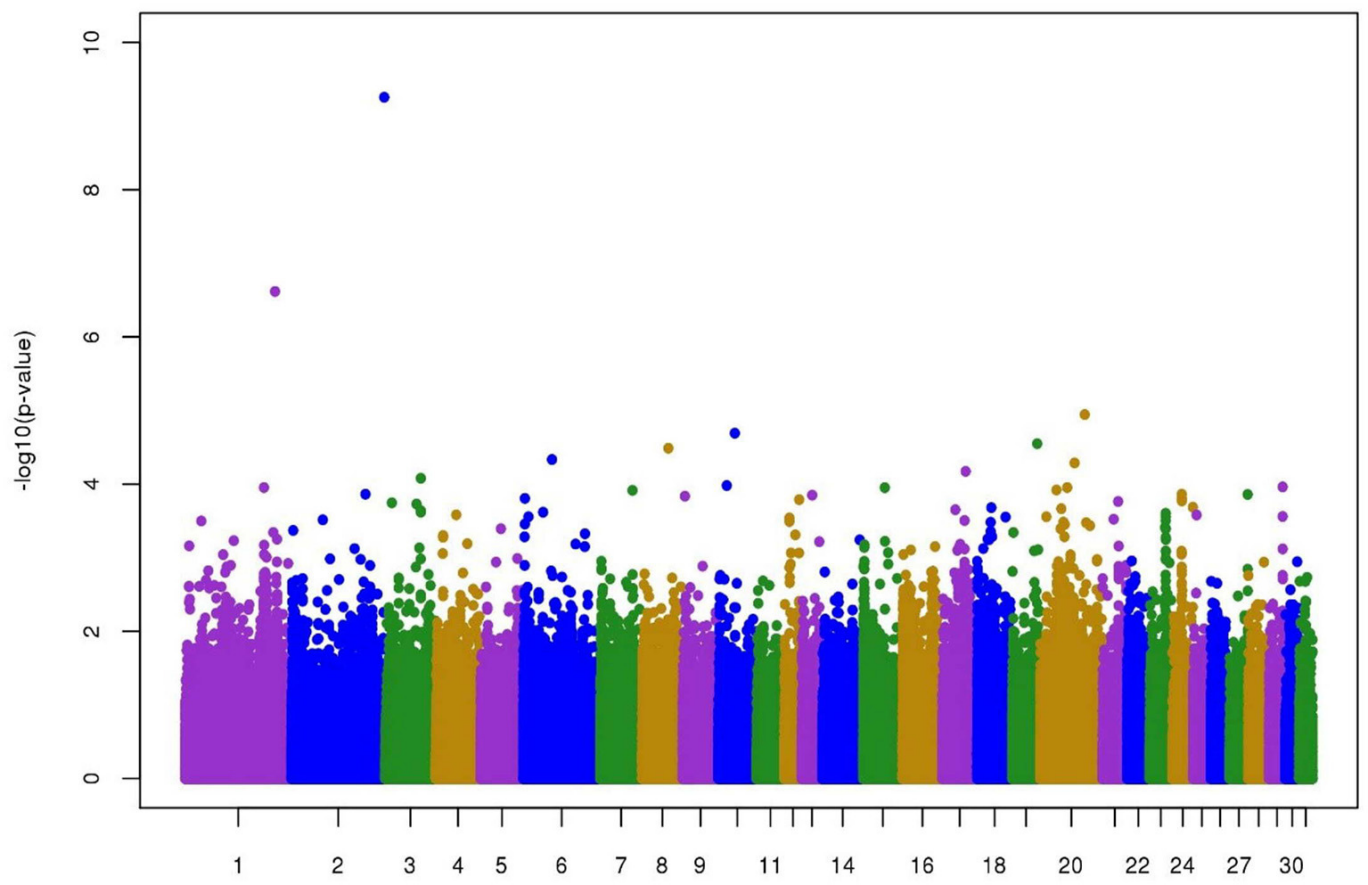
Manhattan plot of -log10(P) of individual SNP effects on longevity in jumping competition.

Figure 2 represents the plot of -log10(P) of the SNP effects on longevity. No clear QTL was found. The two SNPs with -log10(P) higher than 5 were located; one on chromosome 1 173829259 (6.6) and the other on chromosome 2 120452449 (9.3). The MAF for these two SNPs was low and close to the threshold used for quality control, namely 6.7 and 5.3%.

## Discussion

### Accuracy of the Results

Because the data were obtained from official competitions, a large number of unselected horses were used to estimate the breeding values for the functional longevity of stallions on a very long timescale (33 years). This resulted in accurate estimated heritability and breeding values. A much smaller number of horses was used to record gaits. The accuracy for the estimation of variance components for the gait traits was lower due to the small number of progenies from the stallions (mean 3.0). A large number (70%) of horses with gaits data were derived from a sire with a pseudo-phenotype for longevity, which assured accuracy for the genetic correlations, and with a higher number of progenies derived from these sires (4.6). Moreover, the absence of horses with both traits may -to some extent- prevent the confusion between genetic and environmental correlations in case of interaction between genetic and environments effects. Genomics was helpful, with both a large number of horses with genotyped gait data (78%), as well as their sires (64%). The deregression method to obtain longevity pseudo-performances loses information. This consequently limits the degree of accuracy, but it allows association of a nonlinear model with a linear model and linkage of functional longevity to early traits. In addition, using simulated data, Tarres et al. (2006) were able to show the relevance of this method. Only 1,968 of the initial 16,564 horses were retained, but they represented the horses with a large proportion of their progeny in competition. They were hence representative of competition, and this allows numerical problems due to deregression formulae to be avoided.

### Functional Longevity Analysis

Functional longevity analysis has been used in France for a long time. The results shown here are in accordance with previous results, both for variance component estimation and for fixed effects solutions (Ricard and Fournet-Hanocq, 1997; Ricard and Blouin, 2011). The estimated heritability was 0.12, which is close to the previously reported value of 0.08 (Ricard and Blouin, 2011). A better focus on specialized jumping horses, after dropping horses that also performed in eventing and dressage, may explain the higher heritability. These findings are in line with the heritabilities estimated in several countries for jumping horses, as well as for dressage and eventing (Braam et al., 2011; Jonsson et al., 2014a; Posta et al., 2014; Seiero et al., 2016; Sole et al., 2017). In these studies, longevity was measured as the number of years in competition, except for Sole et al. (2017), for whom the criterion was the number of dressage tests, independently of the time spent in competition. In Sole et al. (2017) and Posta et al. (2014), survival analysis was used to analyze longevity, and longevity was corrected for the level of performance as in our study. In Sole et al. (2017) the heritability was 0.20 for longevity in dressage competition, while in Posta et al. (2014) heritability was 0.17 for longevity in jumping competition. In the other studies, survival analysis was not used. The measurement of longevity was the number of years in competition, and it was mathematically transformed to obtain better normality. The most important difference between these studies and our study was that longevity was not corrected for the level of performance. We showed the strong effect of the level of performance on longevity. Thus, when the level of performance is not included in the model, the longevity trait can be a mix of physical resistance and jumping qualities. Therefore, heritability may be higher, corresponding to the heritability of jumping performance. In Sweden, Braam et al. (2011) found a heritability of 0.14 for show jumping, and Jonsson et al. (2014a) found a value of 0.20 when the number of years in competition was counted irrespective of the discipline practiced during the year (jumping, dressage, or eventing). In Denmark, Seiero et al. (2016) found a heritability of 0.11 for jumping horses.

The novel finding of this study in regard to longevity was the absence of major QTLs influencing the trait. For jumping ability, several putative QTLs were found (Schroeder et al., 2012; Brard and Ricard, 2015; Nanaei et al., 2019), but the trait remained largely polygenic, with no major genes found (Stock et al., 2016). The major genes found for health traits related mostly to syndromes that are not relevant to jumping longevity, as they are related to diseases that mostly occur before the horse is able to enter competitions (Finno and Bannasch, 2014), except for certain types of myopathy, such as a mutation in GYS1 that could explain the phenotype (McCue et al., 2008, 2009). But no significant SNPs were found near GYS1. Longevity in jumping competitions is a complex polygenic trait, like jumping ability.

### Correlation With Gaits

Genetic correlation between longevity and gait assessment has also been estimated in Nordic countries. In Denmark and Sweden, young horses (4–5 years old) are judged during Riding Horse Quality Tests (RHQT). Gait scores as well as conformation or rideability are recorded. Genetic correlations between these assessments and the number of years in competition have been estimated. Seiero et al. (2016) found no significant genetic correlation between longevity and correctness of movement or elasticity (-0.15, SE 0.12;−0.13 SE 0.10), but they did find a high positive genetic correlation between longevity and canter 0.56 (SE 0.12). In Sweden, Braam et al. (2011) found an overall positive correlation between temperament gaits 0.29 (SE 0.09) with the number of years in competition for all disciplines (including jumping, dressage, and eventing). Jonsson et al. (2014a) found a more detailed genetic correlation of 0.14 (SE 0.08) for walk, 0.24 (SE 0.07) for trot, and 0.31 (SE 0.07) for overall gaits talent with the same number of years in competition for all disciplines. In these last two studies, they also performed genetic correlation with accumulated lifetime points to measure the jumping competition performance. They found essentially the same genetic correlations between gaits and this lifetime performance. This was not surprising because the genetic correlation between the number of years in competition and lifetime performance (accumulated number of upgrading points during the career) was 0.76 (SE 0.04) (Braam et al., 2011). Studying longevity without correction for the level of performance leads to confusion between aptitude for the competition and physical endurance and the level of health necessary to remain in competition for a long time. Thus, assessment of gaits mostly denotes an overall good impression of the horse for competition rather than a detailed description of which gaits will give rise to physical resistance in competition. This overall good impression has already been proven by the genetic correlations found between these assessments and the health status recorded independently during Riding Horse Quality Tests; RHQT (Jonsson et al., 2014b). These authors found genetic correlations ranging from 0.23 (SE 0.11) to 0.43 (SE 0.10) between walk and trot scores and overall orthopedic health. While one cannot deny the existence of these genetic correlations, they remain difficult to interpret separately from fitness because specifics for the criteria for judging gait are lacking. The evaluation of canter in the context of free jumping, for example, can bias a strong genetic correlation between longevity and canter due to the forward movement required for the jump.

### How to Interpret the Correlations?

The difference with previous results regarding the relationship between gaits and longevity was that the accelerometry provided a description of the gaits and not an assessment of the gaits. We found that (1) lateral activity in trot associated moderately with lower longitudinal activity, higher dorsoventral activity, and higher stride frequency and (2) high dorsoventral activity in canter associated with low stride frequency had a moderate favorable effect on functional longevity. Because the models included velocity and height, activities must be interpreted as residuals necessary to obtain the same gait velocity at a given height. It is difficult to get an understanding at the biological level why lateral loss of activity during trot had a moderate favorable effect on jumping longevity. Perhaps this criterion distinguishes the horse from the traditional expectation about trotting technique suitable for a dressage horse rather than a jumping horse. It has also been proven that the two disciplines belonged to different qualities in sport breeds (Rovere et al., 2015, 2017; Ablondi et al., 2019). For the scoring during young horse testing, no distinction was made between assessment with the objective of a dressage or a jumping career. Correlations obtained in Nordic countries were not able to disentangle these differences. The moderate favorable effect of higher dorsoventral activity in canter was easier to interpret as a natural propensity for the horse to maintain velocity with a lower number of strides and a more bouncing gait assuring less physical damage for the jumping career. We found no genetic correlation between the height at withers and functional longevity. A low genetic positive relationship was found by Jonsson et al. (2014a) between the height at withers and longevity, with rg = 0.19 (SE 0.07), which was in keeping with the genetic correlation they found between height and health status, with rg = 0.32 (SE 0.06) (Jonsson et al., 2014b). From a phenotypic point of view, Ducro et al. (2009) found no significant risk ratio associated with height for jumping longevity, whereas there was a higher risk for the horses that were 25% taller in regard to dressage longevity. Height was not associated with locomotion issues phenotypically in Visser et al. (2014), although it was associated with back pain disorders. Therefore, there is no particular problem relating to height for selection of a durable horse for jumping competition in the range of the actual average size of Selle Français horses (in this sample, the mean = 166.7 and the SD = 4.2 from 151 to 181).

We found no significant genetic correlation in the same sample with jumping competition results, except with the PC3 for canter linked to longitudinal activity (Ricard et al., 2020): a higher activity was linked to a worse performance (rg = −0.22, SE 0.10). Therefore, it was rather surprising to find two components genetically moderately linked to functional longevity with a higher absolute correlation. These components are different from the one linked to jumping performance, but it was expected by the study of longevity already corrected for the level of performance. The use of such results for selection purposes should firstly be to be able to better describe gaits, even in assessments during breeding shows in France, in order to be able to qualify both aptitude and correct traits for indirect selection of functional longevity.

## Conclusion

Improving functional longevity is an important current objective for breeders of sport horses and the Selle Français Stud-book, as it directly impacts the emotional and economic investment of horse owners and riders as well as the welfare of the horses in competition. Such improvement requires an indirect selection of traits observable in young horses due to the delay in obtaining information regarding the longevity of stallions or mares. This, in particular, requires a precise and objective assessment and description of these early traits. The study provides a better understanding of the traditional relationship between longevity in jumping competition and early gait assessment. Firstly, because we studied functional longevity instead of a lifetime measure of performance and secondly because gaits were described by objective accelerometer measurements. A specific methodology was necessary to link nonlinear longevity traits to linear gaits traits. Genomics was used to seek potential major genes and to increase the accuracy of the results. Unfortunately, the genomic study did not reveal major QTLs for longevity that could facilitate precocious selection. We found a moderate genetic correlation between longevity in jumping competition and two characteristics of gaits: lateral activity at trot and dorsoventral activity, and low stride frequency at canter. The biological explanation for this is not obvious. Therefore, we must focus on the understanding of the biomechanics of the gaits related to longevity before implementing it in a selection plan. Future studies are required in this regard. From a practical point of view, the use of accelerometry in the routine of breeding shows can be relatively easy. The accelerometer is a small, light device that is attached to the strap or to a surcoat and the measurements are therefore easy to do as it does not change the horse's harnessing habit. The difficulties lie more in the development of dedicated software for instant results. The French stud book already engaged a collaboration with a saddler firm to adapt device on surcingle to use it in regularly in breeding shows for free jumping.

## Data Availability Statement

The data used in this study are the property of the Selle Français Stud-Book and the owners of the horses. There are restrictions on the availability of this data and it is not publicly available. Access to the data may be authorized by the partners of the SoGen project within the framework of which they were collected, on request from the authors. Requests to access these datasets should be directed to Manon Dugué, manon.dugue@inrae.fr.

## Ethics Statement

Ethical review and approval was not required for the animal study because the measurements on the horses took place during official competitions with the agreement of the owners. Written informed consent was obtained from the owners for the participation of their animals in this study.

## Author Contributions

MD and AR carried out the statistical study. HC performed the imputations for the genotyping. BDSP carried out the phenotyping. SD and AR coordinated the study. The article was written by MD and AR. All authors contributed to the article and approved the submitted version.

## Conflict of Interest

The authors declare that the research was conducted in the absence of any commercial or financial relationships that could be construed as a potential conflict of interest.
