# Supplementary material for: Genomic Correlations Between the Gaits of Young Horses Measured by Accelerometry and Functional Longevity in Jumping Competition
Source: Front Genet. 2021 Jan 29;12:619947. doi: 10.3389/fgene.2021.619947 (PMC7879571; doi:10.3389/fgene.2021.619947)
Supplement: Supplementary file 1 [file Table_1.DOCX]

**Table. Descriptive statistics of the accelerometry variables** of the sample of young four or five years old jumping horses (n=1477) **(mean ± SD)**

| Gait^a^ | MT | WT | MC | WC | W |
| --- | --- | --- | --- | --- | --- |
| N | 1398 | 1451 | 1372 | 1357 | 1436 |
| Velocity (m/s) | 4.35 ± 0.43 | 3.57 ± 0.43 | 6.22 ± 0.7 | 5.20 ± 0.58 | 1.77 ± 0.2 |
| Stride Frequency (/s) | 1.46 ± 0.08 | 1.37 ± 0.07 | 1.73 ± 0.08 | 1.68 ± 0.07 | 0.91 ± 0.06 |
| regularity | 312.0 ± 47.0 | 348.4 ± 36.2 | 113.3 ± 24.8 | 127.2 ± 22.4 | 178.3 ± 47.3 |
| symmetry | 5.45 ± 0.20 | 5.50 ± 0.20 |  |  | 5.30 ± 0.26 |
| dorsoventral displacement (cm) | 10.38 ± 1.89 | 10.75 ± 1.85 | 21.36 ± 2.45 | 20.50 ± 2.14 | 3.97 ± 1.46 |
| dorsoventral activity (W/kg) | 19.45 ± 3.30 | 14.73 ± 3.19 | 2.93 ± 0.18 | 2.70 ± 0.17 | -0.22 ± 0.46 |
| longitudinal activity (W/kg) | 2.36 ± 0.32 | 2.14 ± 0.31 | 2.96 ± 0.46 | 2.46 ± 0.42 | 0.96 ± 0.40 |
| latteral activity (W/kg) | 1.34 ± 0.33 | 0.98 ± 0.35 | 1.95 ± 0.33 | 1.68 ± 0.31 | 0.24 ± 0.35 |

^a^ WT = working trot, MT = Medium Trot, WC = Working canter, MC = medium canter, W = walk.
