# Supplementary material for: Genomic Correlations Between the Gaits of Young Horses Measured by Accelerometry and Functional Longevity in Jumping Competition
Source: Front Genet. 2021 Jan 29;12:619947. doi: 10.3389/fgene.2021.619947 (PMC7879571; doi:10.3389/fgene.2021.619947)
Supplement: Supplementary file 2 [file Table_2.DOCX]

Supplementary Material

# Calculation of cleaned $\boldsymbol{R}$s

## Calculation of $\boldsymbol{R}$ without ascendants

$R_{X+Y}=\frac{R_{X}+R_{Y}-2R_{X}R_{Y}}{1-R_{X}R_{Y}}$ (Harris Johnson, 1998).

Horse A is a parent of horse B. We denote X as the information brought to the $R$ of horse A by its relatives other than horse B, and we denote Y as the information brought to the $R$ of horse B by his relatives other than horse A. The formula becomes

$R_{A,X+Y}=\frac{R_{A,X}+R_{A,Y}-2R_{A,X}R_{A,Y}}{1-R_{A,X}R_{A,Y}}$for horse A

and

$R_{B,X+Y}=\frac{R_{B,X}+R_{B,Y}-2R_{B,X}R_{B,Y}}{1-R_{B,X}R_{B,Y}}$for horse B.

However,

$R_{B,X}=\frac{1}{4}R_{A,X}$ and $R_{A,Y}=\frac{1}{4}R_{B,Y}.$

To find the $R$ of horse B without the information provided by its parent A, i.e.$, R_{B,Y}$, we solve the following nonlinear system:

$$\left\{ \begin{aligned} R_{A,X+Y}=\frac{R_{A,X}+0,25R_{B,Y}-0,5R_{A,X}R_{B,Y}}{1-0,25R_{A,X}R_{B,Y}} \\ R_{B,X+Y}=\frac{0,25R_{A,X}+R_{B,Y}-0,5R_{A,X}R_{B,Y}}{1-0,25R_{A,X}R_{B,Y}} \end{aligned} \right.$$

Likewise, new $R$s are calculated for horses with a sire and a sire and a maternal grandsire.

## Calculation of $\boldsymbol{R}$ without products

Once we have cleaned the $R$s of the information provided by their parents included in the group of stallions, we must delete the information provided by their products included in this group. To find the $R$ of horse A without the information provided by its descendant B, i.e., $R_{A,X}$, we calculate

$R_{A,X+Y}=\frac{R_{A,X}+R_{A,Y}-2R_{A,X}R_{A,Y}}{1-R_{A,X}R_{A,Y}}$with $R_{A,Y}=\frac{1}{4}R_{B,Y}$

*i.e.,*

$$R_{A,X}=\frac{R_{A,X+Y}-0,25R_{B,Y}}{1+0,25R_{A,X+Y}R_{B,Y}-0,5R_{B,Y}}.$$

Likewise, new $R$s are calculated for horses with a grandson.

The final $R$ retained is $R':=R_{A,X}.$
